# Hospital Records

**Published:** 1866-11

**Authors:** 


### HOSPITAL RECORDS.

In the October number of the Journal, we commenced the publication of Hospital reports from the Army of Tennessee. They will be found in this and subsequent numbers, until the material is exhausted, from which these statistics are obtained.

As a source of information to many who may feel an interest in examining a record of deceased soldiers in the Confederate Army of Tennessee, as well as in a scientific point of view, we hope to make them useful.

Many of such records having been destroyed, or placed beyond the control of those who seek their publication, it is but justice to the bereaved friends, and to the memory of the dead, that all available material be used for this purpose. In their publication, valuable medical statistics are afforded the scientific inquirer, and as such will be found useful to our readers.

Below will be found reports to 31st December, 1861, as furnished by our valuable contributor, Prof. S. H. Stout:

*Statement of Deaths of Confederate Soldiers at and near Pensacola, Fla., During October*, 1861.

William Calvert pvt. 10th Miss.

J. H. Byrum, " 9th "

Joel Williams, " 7th Ala.

H. H. Baroum, " 7th Miss.

J. C. Cole, pvt. Georgia Battalion.

W. E. Caldwell, pvt. 9th Miss.

Thomas Berry, " 10th "

J. D. Sloan, " " "

H. A. Berryhill, " " "

R. W. Wheeler, " 9th Miss.

W. Bailey, pvt. 1st Ala.

R. D. Sprowls, pvt. Geo. Bat.

A. J. Martin, " 10th Miss.

W. C. Weems, " " "

James Davis, " La. Inft.

John Smith, " 1st Ala.

Robert J. Hayes, pvt. 5th Georgia.

——— Thompson, pvt. 1st Fla.

William Smith, " " "

J. D. T. Warters, " " "

E. T. Boylston, Sergt. " "

James Jourdan, pvt. Marines.

James M. Watson, pvt. 5th Geo.

A. R. Carothen, pvt. 5th Geo.

James H. Biddow, Sergt. 5th Geo.

L. C. Hollingsworth, Sergt. Geo. and Miss.

Thomas Duffie, Corp. 1st La. Inft.

W. L. Johnson, pvt. 5th Geo.

Walter Tuggle, " 5th Ala.

Newman Knox, " 7th "

Lewellyn Nelms Lieut. 5th Geo.

James H. Beddo, Sergt. " "

Chas. H. Caton, Corp. " "

John Cluxton, pvt. La. Inft.

Stephen K. Crawley, pvt. 5th Geo.

Henry Tappin, pvt. La. Infantry.

William Holbrook, pvt. La. Inft.

James Curley, " " "

—— Ferguson, " 7th Ala.

William H. McKinnis, pvt. 1st Fla.

Richard Bradford, Capt. " "

William R. Routh, Sergt. " "

Henry Tillinghast, pvt. " "

Robt. Hale, " " "

James F. Jones, pvt. 5th Geo.

Damascus L. Codep, pvt. 5th Geo.

Fred. F. Cook, " " "

Jos. F. Adams, " " "

John Staunton, " " "

Robt. Hays, " " "

James Smith, " " "

Reuben W. Stewart, Corp. 5th Geo.

Thos. W. Everett, pvt. " "

Noah Sinclair, pvt. 9th Miss.

RECAPITULATION.

| | |
|---|---|
| Febris Typhoides | 13 |
| " Remittens | 1 |
| " Congestiva | 3 |
| Pneumonia | 1 |
| Pneumonia Typhoides | 1 |
| Rubeola | 1 |
| Dysenteria Acuta | 2 |
| Ascites | 1 |
| Debilitas | 1 |
| Vulnus Sclopeticum | 29 |
| Unknown | 1 |
| Total | 54 |

SUMMARY, OCT., 1861.

| | | |
|---|---|---|
| Remaining from last report | | 748 |
| Received during the month | | 2,465 |
| Aggregate | | 3,213 |
| Sent to General Aospital | 333 | |
| Returned to duty | 1,611 | |
| On Furlough | 110 | |
| Discharged the service | 56 | |
| Deserted | 8 | |
| Died | 54 | |
| Remaining sick | .574 | |
| Convalescent | .467 | |
| | —1,041 | |
| Total Remaining | | 3,213 |
| Mean strength—Officers | 372 | |
| " " —Enlisted men | 6,423 | |
| Total | 6,795 | |
| Ratio of Deaths per 1,000, mean strength | | 7.947 |

*Statement of Deaths of Confederate Soldiers at and near Pensacola, Fla., for November*, 1861.

W. S. Moore, pvt. 7th Ala.

J. W. White, " 9th Miss.

S. C. Tucker, " 1st Ala.

J. T. Lawrison, pvt. 9th Miss.

J. C. Hodge, pvt. 1st Ala.

H. A. Parker pvt. 1st Fl .

John Moran, pvt. La. Inft.

M. S. Tisdell, pvt. 17th Ala.

George Beasley, pvt. 36th Georgia.

E. Ripper, pvt. 17th Ala.

Micle McGuire, pvt. 17th Ala.

G. S. Ducknortte, pvt. 9th Miss.

H. Neddermeyer, pvt. La. Inft.

Daniel Farrell, pvt. 1st. Ala.

Walter Handley, pvt. 1st Ala.

J. H. Williams pvt. Watt's Regt.

Wm. McClinton, pvt. Dra's Regt.

Pleasant Moore, pvt. Wheeler's Regt.

J. M. Turbeville, pvt. Loomis's Bat.

W. T. Powell, pvt. Loomis's Bat.

Jas. H. Higgins, pvt. Wheeler's Regt.

Wm. Russell, pvt. Wheeler's Regt.

W. Barlow, Corp. Beck's Regt.

Joseph King, pvt. 2d Ala.

RECAPITULATION.

| | |
|---|---|
| Febris Typhoides | 6 |
| Pneumonia | 6 |
| Bronchitis | 1 |
| Dysenteria Acuta | 5 |
| Dysenteria Chronica | 1 |
| Scorbutus | 2 |
| Disease of Heart | 1 |
| Rupture of Bladder | 1 |
| Vulnus Laceratum | 1 |
| Vulnus Sclopeticum | 1 |
| Total | 35 |

In addition to the above, by sentence of General Court Martial, two men of the 10th Mississippi Regiment, were shot to death on the 8th of November, 1861.

SUMMARY FOR NOV. 1861.

| | | | |
|---|---|---|---|
| Remaining from last report | | | 1,041 |
| Received during the month | | | 2,613 |
| Aggregate | | | 3,654 |
| Sent to General Hospital | | 206 | |
| Returned to duty | | 2,451 | |
| On Furlough | | 49 | |
| Discharged the service | | 75 | |
| Deserted | | 3 | |
| Died | | 25 | |
| Remaining sick | 446 | | |
| Remaining convalescent | 399 | | |
| | | 845 | |
| Total remaining | | | 3,654 |
| Mean strength—Officers | | 418 | |
| " " —Enlisted men | | 7,216 | |
| Total | | 7,634 | |
| Ratio of deaths per 1,000 mean strength | | | 3.274 |

*Statement of Deaths of Confederate Soldiers at and near Pensacola Florida, During the month of December*, 1861.

W. J. Williamson, pvt. 17th Ala.

W. C. Tompson, pvt. 17th Ala.

Jas. W. Shearer, pvt. 0th Miss.

Thos. Halcombe, pvt. 1st Ala.

T. Philpot, pvt. 17th Ala.

L. J. Parker, pvt. 24th Miss.

J. M. Chamblic, pvt. 5th Miss.

John Wallace, pvt. 17th Ala.

T. R. Rosdick, pvt. 17th Ala.

H. B. Moore, pvt. 9th Miss.

John M. Sheppard, pvt. 17th Ala.

M. G. Farrow, pvt. 17th Ala.

Tho. Porterfield, pvt. 9th Miss.

S. Alderman, pvt. 5th Miss.

Jas. A. Parks, pvt. 17th Ala.

T. J. Saunders, pvt. 17th Ala.

Som. Vaughn, pvt. 17th Ala.

N. Y. Ramsey, pvt. 17th Ala.

J. H. Johnson, pvt. 17th Ala.

M. G. Pegler, pvt. 17th Ala.

John Glenn, pvt. 17th Ala.

W. Cummings, pvt. 17th Ala.

Wm. Jackson, pvt. 17th Ala.

James Falkner, pvt. 17th Ala.

Wm. F. De Fee, pvt. 17th Ala.

Geo. Hawthorne, pvt. 1st Ala.

A. R. Allen, pvt. 17th Ala.

J. M. Wright, pvt. 17th Ala.

Wm. Lee, pvt. 17th Ala.

F. B. Peck, pvt. 17th Ala.

Dan Swift, pvt. La. Inft.

T. B. Parham, pvt. 5th Miss.

M. W. Smart, pvt. 17th Ala.

M. J. Blackburn, pvt. 17th Ala.

A. G. Drummond, Cooper's Rifles.

P. Crawford, pvt. 36th Geo.

W. H. Scott, Capt. La. Inft.

Lewis Harrell, pvt. 5th Miss.

Thomas Walker, pvt. 8th Miss.

Isaac Barnes, pvt. 5th Miss.

S. Scarborough, pvt. 8th Miss.

John Pate, pvt. 8th Miss.

Wil. T. Mickey, pvt. 10th Miss.

RECAPITULATION.

| | |
|---|---|
| Febris Typhoides | 16 |
| Febris Remittens | 5 |
| Febris Intermittens | 1 |
| Pneumonia | 8 |
| Pneumonia Typhoides | 2 |
| Rubeola | 1 |
| Dysenteria Acuta | 6 |
| Diarrhœa | 1 |
| Bronchitis Chronica | 1 |
| Cerebritis | 1 |
| Concusio Cerebri | 1 |
| Total | 43 |

NOTE.—The report for December, 1861, is incomplete. No surgeon was attached to the 24th Mississippi for a part of the month. This regiment suffered from an epidemic—Rubeola followed by Typhoid Fever.

SUMMARY FOR DEC. 1861.

| | | | |
|---|---|---|---|
| Remaining from last report | | | 845 |
| Received during the month | | | 2,553 |
| Aggragate | | | 2,398 |
| Sent to General Hospital | | 294 | |
| Returned to duty | | 973 | |
| On Furlough | | 67 | |
| Discharged the service | | 90 | |
| Deserted | | 00 | |
| Died | | 43 | |
| Remaining sick | 603 | | |
| Remaining convalescent | 328 | | |
| | —931 | | |
| Total remaining | | | —2,398 |
| Mean strength—Officers | | 432 | |
| " " —Enlisted men | | 7,203 | |
| Total | | 7,635 | |
| Ratio of deaths to 1,000 of mean strength | | | 5.646 |

GENERAL SUMMARY FOR THE QUARTER ENDING DEC. 31ST, 1861.

| | | | |
|---|---|---|---|
| Remaining at last report | | | 748 |
| Received during tne Quarter | | | 7,631 |
| Aggregate | | | 7,379 |
| Sent to General Hospital | | 833 | |
| Returned to duty | | 5,035 | |
| On Furlough | | 226 | |
| Discharged the service | | 2{3}1 | |
| Deserted | | 11 | |
| Died | | 122 | |
| Remaining sick | 603 | | |
| Remaining convalescent | 328 | | |
| | | — 913 | |
| Total remaining | | | —7,379 |

